# Quality Improvement Project to Improve Adherence to Best Practices to Decrease Incidence of Necrotizing Enterocolitis in Preterm Infants

**DOI:** 10.3390/children12020176

**Published:** 2025-01-30

**Authors:** Ahreen Allana, Sidra Bashir, Ivan Hand

**Affiliations:** 1 Department of Pediatrics, NYC Health & Hospitals/Kings County, Brooklyn, NY 11203, USA; ahreen.allana@utsouthwestern.edu (A.A.); sidra.bashir@downstate.edu (S.B.); 2 Department of Pediatrics, SUNY-Downstate College of Medicine, Brooklyn, NY 11203, USA

**Keywords:** NICU, necrotizing enterocolitis, NEC, preterm, PDSA cycle

## Abstract

**Background/Objectives:** Necrotizing enterocolitis (NEC) is one of the most devastating gastrointestinal emergencies in preterm infants. This quality improvement (QI) project aimed to increase the utilization of accepted evidence-based practices in our neonatal intensive care unit (NICU) to ultimately decrease the incidence of NEC in our level III NICU. **Methods:** Our QI team implemented a bundle of nine of these evidenced-based practices for NEC prevention and disseminated information among the NICU team. Items in the bundle included delayed cord clamping, parental education on the importance of breast milk, obtaining early consent for donor breast milk, adherence to the unit’s feeding protocol, avoiding routine gastric residual checks, the discontinuation of antibiotics at 48 h once blood cultures were negative, restricting the use of antacids, nasogastric tube (NGT) replacement every 72 h and the removal of central lines once a feeding volume of 100 mL/kg/day was attained. The baseline incidence of clinically proven NEC was found to be 7% at the start of the intervention. We conducted two Plan-Do-Study-Act (PDSA) cycles over a 2-year period from 1 January 2021 to 31 December 2022. **Results:** There were 74 infants who met the inclusion criteria of being <1500 g and/or at <32 weeks of gestation. The adherence to our process measures improved over the course of our two PDSA cycles from 78% adherence to 91.6%, *p* < 0.05. The incidence of NEC decreased from 7% to 5.3% following the first PDSA cycle, a 24% reduction. Following the second PDSA cycle, the incidence decreased even further from 5.3% to 2.8%, a 60% reduction from baseline, although this was not statistically significant due to the small sample size. **Conclusions:** In this QI initiative, we achieved improved adherence to several evidence-based interventions over a two-year period with the aim of reducing the incidence of NEC at our institution.

## 1. Introduction

Necrotizing enterocolitis (NEC) is one of the most devastating gastrointestinal emergencies in preterm infants. The incidence of NEC has been reported to be 2–7.5% in very-low-birth-weight (VLBW) infants, contributing to significant morbidity and mortality in neonatal intensive care units (NICUs) [1,2,3]. The incidence varies inversely with gestational age and birth weight, with studies demonstrating a five-fold greater risk among infants < 28 weeks gestational age (GA) and weighing less than 1000 g [4]. The mortality associated with NEC has been estimated to range between 20 and 30% among infants requiring surgery [5]. Amongst survivors, complications include neurodevelopmental impairment and gastrointestinal comorbidities requiring long-term multidisciplinary rehabilitation [6]. The high mortality and morbidity rates of NEC and its consequent impact on healthcare costs make it one of the most expensive neonatal pathologies, incurring an estimated annual financial burden of USD 500 million–1 billion in the United States [7].

Despite advancements in neonatal care with the increasing survival rates of preterm infants at younger gestational ages, the multifaceted pathophysiology of NEC makes it a diagnostic and therapeutic challenge. Over the past decade, there has been substantial progress in research aimed at understanding the pathophysiology of NEC. Studies in animal models suggest a multifactorial etiology, with the concurrent presence of intestinal and immunological immaturity that triggers an exaggerated host immune response, subsequently leading to microbial dysbiosis and the overgrowth of pathogenic bacteria [8,9,10]. The consequent intestinal injury can progress to infection and necrosis, multi-organ failure and death. Even though the multifactorial nature of NEC limits the successful implementation of treatment and preventative strategies, the incidence of NEC has progressively declined over the past decade, partly due to quality improvement initiatives that target various predisposing factors [11].

The implementation of these quality improvement initiatives at various institutions in the US and around the world has led to a substantial reduction in the global incidence of NEC [11]. As summarized by Patel et al., adherence to best practice guidelines, driven by PDSA (Plan-Do-Study-Act) cycles and continuous data monitoring, has demonstrated improved outcomes in VLBW infants, including a decreased incidence and severity of NEC [12]. These quality improvement measures include best practice guidelines such as standardizing enteral feeding guidelines, exclusive mother’s own milk (MOM) feeds, the use of donor human milk (DHM) when MOM is not available, minimizing the duration of antibiotics, avoiding the use of antacids and implementing delayed cord clamping after birth. Other practices that lack a high certainty of evidence include the avoidance of anemia, feeding guidelines during blood transfusions and the use of probiotics [12].

Adherence to standardized feeding protocols has demonstrated a decrease in the incidence of NEC in many units despite variations in the rates of feeding advancement and fortification practices. Some of the mechanisms that may play a role in these feeding protocols include consistent care practice and the avoidance of rapid increases in feeding volumes [13]. Structured feeding regimens and exclusive human milk (HM) feeds remain essential components of most quality improvement initiatives [14,15,16,17,18,19]. Statistically significant decreases in the NEC rate and mortality have been achieved with the implementation of comprehensive nutritional bundles and strategies to increase the use of HM [16,20]. Similar findings have been noted with the initiation of early human milk feeds in addition to the incorporation of conservative feeding guidelines during blood transfusions and patent ductus arteriosis (PDA) treatment, the restriction of H2 antagonist use, antibiotic stewardship [21,22,23], the early removal of central lines and delayed cord clamping [18,24,25]. Reductions in NEC rates have also been found with quality improvement measures that focused on optimizing the gut microbiome with the use of probiotics and human milk-derived fortifiers [26,27,28]. However, given the lack of US Food and Drug Administration approval for probiotics, their use is not a standard recommended practice.

In summary, the implementation of evidence-based interventions has demonstrated a successful and sustainable reduction in the incidence of NEC and associated morbidity and mortality in VLBW infants across multiple centers. Given NEC’s multifactorial pathogenesis, it is crucial to employ a combination of preventative strategies that target multiple pathways. We conducted a QI project based on improving adherence to a bundle of clinically proven interventions with the ultimate goal of decreasing the incidence of NEC by >20% over a 2-year period. The baseline incidence of NEC was 7% at the start of the initiative, based on an analysis of our unit data from 1 January 2019 to 30 June 2020.

## 2. Materials and Methods

### 2.1. Context and Setting

This QI project was conducted at the 30-bed level III NICU of NYC Health + Hospitals/Kings County. The Institutional Review Board at the SUNY Downstate Health Sciences University provides oversight for all human subject research in affiliated facilities. This QI project was not considered human subject research and received exemption from the Institutional Review Board.

### 2.2. Study Population and Period

All inborn and externally transferred infants < 32 weeks gestational age (GA) and/or <1500 g birth weight (BW) were included. Infants with congenital anomalies were excluded. The intervention period was from 1 January 2021 to 31 December 2022. We aimed to perform at least 2 PDSA cycles lasting 1 year each over the 2-year study period. The baseline incidence of NEC was 7% during the prior 12-month period of 1 January 2020 to 31 December 2020.

Our population was predominantly African American with more than 95% of mothers identifying themselves as non-Hispanic black. It is worthwhile to mention that the overall rate of exclusive breastfeeding in our study population was <20%, which is lower than the national average [29]. Donor human milk (DHM) was introduced at our institution in 2016, and its use was associated with the earlier initiation of enteral nutrition, faster return to birth weight (BW) and a statistically significant decline in the incidence of NEC [30]. However, this reduction was not fully sustainable, largely owing to the inconsistent use of DHM as well as misconceptions regarding its use amongst mothers. This necessitated the NEC prevention bundle that aimed to bring about a long-term, sustainable decline in the incidence of NEC and associated mortality.

### 2.3. Key Definitions

Clinical NEC was defined as stage II or higher based on the Modified Bell Staging Criteria [31]. Per this definition, a clinical diagnosis of NEC was made based on the presence of at least 1 physical finding (abdominal distension, gastric retention, absent bowel sounds, emesis or occult/gross blood in stool) in addition to at least 1 radiographic finding (pneumatosis intestinalis, pneumoperitoneum or portal venous gas). All imaging studies were reviewed by our clinical radiologists as part of their normal practice. NEC was diagnosed by either using this clinical criterion or as evidenced by direct intestinal observation during surgery.

### 2.4. Key Driver Interventions

**Figure 1:**
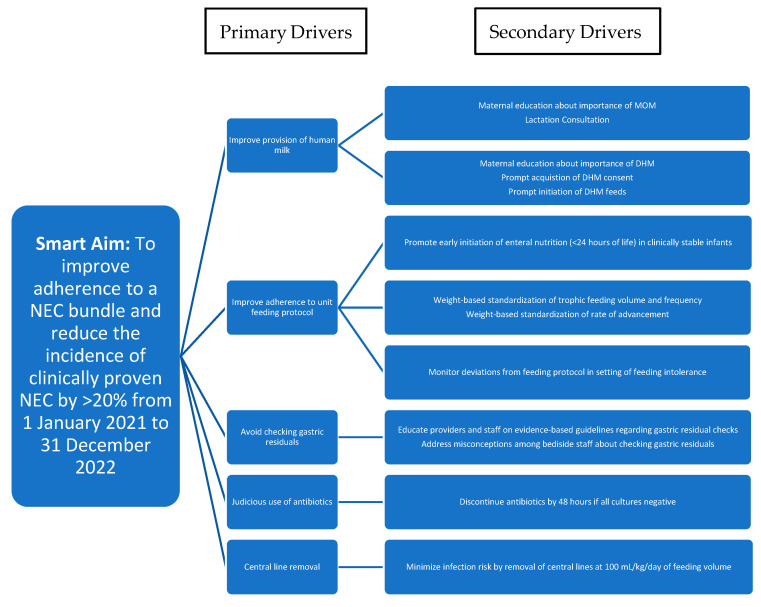
Key driver diagram summarizing interventions driving changes aimed at achieving specific aim of NEC reduction by >20% over 2-year period.

The QI team comprised neonatologists, neonatal fellows, pediatric residents and neonatal nurses. Following a literature review and the determination of the baseline incidence of NEC, the QI team built a driver diagram to achieve our aim of a 20% reduction in clinically proven NEC. Our driver diagram consisted of the primary drivers which directly affect our aim as well as the secondary drivers, which were our interventions to achieve our primary drivers. The key driver diagram (Figure 1) and QI model for improvement were then used to build the NEC prevention bundle that comprised a 9-point checklist. Items on the checklist included 1. delayed cord clamping; 2. approaching and educating parents about the importance of MOM; 3. seeking early consent for DHM; 4. adherence to the unit’s feeding protocol; 5. avoiding a gastric residual check if the infant was asymptomatic; 6. the prompt discontinuation of antibiotics at 48 h once blood cultures were negative; 7. restricting the use of antacids; 8. nasogastric tube (NGT) replacement every 72 h and 9. the removal of central lines once a feeding volume of 100 mL/kg/day was attained.

The bundle was disseminated across the NICU, and interactive educational sessions were conducted for the primary medical team monthly. This multidisciplinary team comprised pediatric residents, nurse practitioners and pediatric hospitalists who provide direct clinical care for all infants in the unit. The team also included neonatal fellows and attendings who provided direct supervision and oversight of the first call providers, as well as neonatal nurses, pharmacists, nutritionists and lactational consultants. The educational sessions were repeated every month at the start of the resident rotation block. For infants who met the inclusion criteria, the NEC bundle was displayed on the infant incubator in the form of a checklist. The bedside RN was responsible for checking off the items as they were completed. After eligible infants were identified and enrolled, the investigators provided additional education to the bedside nursing staff and medical providers who were directly involved in patient care. The investigators also checked in with these individuals on a bi-weekly basis to monitor adherence to the primary drivers of the protocol, answer questions, address misconceptions and identify reasons for non-compliance.

### 2.5. Improving the Provision of MOM

This intervention aimed to improve breast milk supply immediately after delivery. We found that the rate of exclusive breastfeeding in our preterm infants declined after the introduction of DHM. As part of this QI initiative, we reinforced strategies to promote exclusive breastfeeding. The medical team was encouraged to seek prompt lactation consults to promote early pumping and expression and educate mothers about the importance of colostrum and the protective benefits of MOM.

### 2.6. DHM Consent and Prompt Initiation of Enteral Nutrition

The key hallmark of this intervention was to obtain consent for donor human milk as soon as possible after delivery to allow for the early initiation of enteral feeds if MOM was not available. The providers were responsible for obtaining consent for DHM. They also provided education about the importance of human milk as outlined in the consent form and addressed any maternal misconceptions if present.

### 2.7. Adherence to Unit Feeding Protocol

The neonatal feeding protocol at the Kings County Hospital NICU was established in 2016. This protocol is based on evidence-based recommendations about the initiation and advancement of feeding volumes as demonstrated by past research. The feeding volume and frequency are based on infant weight, with smaller infants receiving smaller volumes less frequently than infants who weigh > 1000 g. The initial feeding volume of “trophic feeds” for clinically stable infants < 1000 g is 10 mL/kg/day for the first 5 days of life followed by an increase of 15–20 mL/kg/day. Infants between 1000 and 1500 g are started on feeds at 10 mL/kg/day when clinically stable, with increments of 15–20 mL/kg/day until the attainment of full feeding volume at 140 mL/kg/day. Feeds are fortified to 24 kcal/ounce when the infant receives 60% of the total intake enterally. This weight-specific feeding advancement chart was kept at the bedside, and copies were provided to the clinicians for review during daily multidisciplinary bedside rounds.

### 2.8. Guidelines Regarding Use of Antibiotics, Antacids, Central Lines and Enteral Tubes

These interventions reinforced policies that aimed to limit the risk of infection via the immediate removal of central lines in clinically stable infants once a feeding volume of 100 mL/kg/day was achieved, and NGT replacement was conducted every 72 h. We also sought to restrict the use of antacids and avoid the routine checking of gastric residuals following reports of their increased association with infection, NEC and mortality [32,33,34].

### 2.9. Quality Improvement Measures

Infants who fulfilled the inclusion criteria were identified by 2 independent investigators. The medical records of enrolled patients were reviewed bi-weekly to determine compliance with the bundle and development of NEC. For infants who developed suspected or proven NEC, the investigators reviewed the clinical and radiological criteria to ensure that only infants with NEC stage II or higher were included. These investigators also checked in with the primary team and bedside staff of enrolled infants twice weekly to monitor compliance with checklist items. They identified and addressed any barriers to successful implementation in real time. Enrolled infants were followed bi-weekly until discharge. The primary outcome measure was the incidence of clinically proven NEC. The balancing measure was adherence to checklist items.

## 3. Results

The baseline incidence of clinically proven NEC was found to be 7% at the start of the intervention. We conducted two PDSA cycles over a 2-year period from 1 January 2021 to 31 December 2022, aiming to improve our adherence to best practices in reducing the incidence of NEC. There were 74 infants who met the inclusion criteria with demographic data included in Table 1.

**Figure 2:**
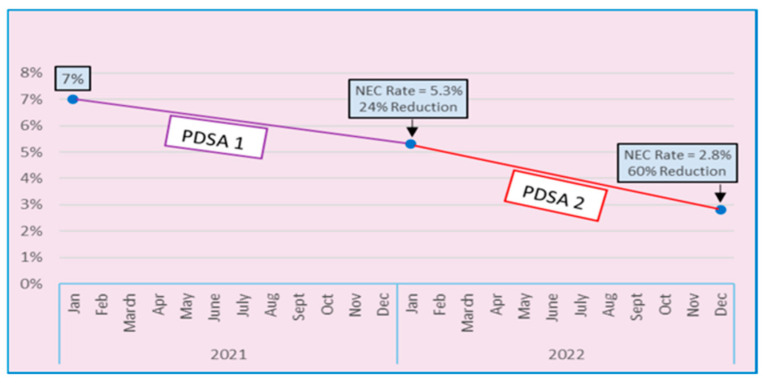
Incidence of NEC over study period.

Figure 2 summarizes the primary outcome. The incidence of NEC decreased from 7% to 5.3% following the first PDSA cycle, a 24% reduction. There were 2 cases of NEC among 38 patients during the first PDSA cycle, 1 requiring surgery and 1 treated with peritoneal drainage. Following the second PDSA cycle, the incidence decreased even further from 5.3% to 2.8%, a 60% reduction from baseline. This represented 1 case of NEC among 36 patients, and it was treated with peritoneal drainage.

Table 2 summarizes the balancing measure of adherence to checklist data. We studied five interventions over the study period. These included the following: 1. increasing MOM provision and obtaining DHM consent; 2. adherence to the unit’s feeding protocol; 3. avoiding gastric residual checks; 4. antibiotic discontinuation after 48 h once all cultures were negative and 5. central line removal in clinically stable infants once a feeding volume of 100 mL/kg/day was attained. We did not monitor compliance with some process measures such as nasogastric tube replacement at 72 h or avoiding antacid use because these practices are universally observed in our NICU. The adherence rates to our process measure improved for all five outcomes following the second PDSA cycle. In addition, a statistically significant improvement was observed with the timely removal of central lines, *p* = 0.023. These changes likely contributed to the 48% decline in the NEC rate observed with PDSA cycle 2.

The continued education of the medical team and nursing staff helped us improve the adherence rates between the two PDSA cycles. Our overall adherence to the NEC bundle improved from 78% after the first PDSA cycle to 91.6%, *p* < 0.05, after the second cycle. During the first PDSA cycle, we observed that deviation from the feeding protocol predominantly occurred in the setting of feeding intolerance. Attempts to mitigate this were made on a case-by-case basis by evaluating the infant’s condition and obtaining information about medical decision making directly from the primary team. For clinically stable infants, once NEC was ruled out, the QI team encouraged the primary team to return to the feeding protocol as soon as deemed appropriate by the attending neonatologist. The decision as to what constitutes clinical stability and the feeding volume upon re-starting feeds was left to the discretion of the attending neonatologist.

The low compliance with other interventions such as central line removal and avoiding gastric residual checks during the first PDSA cycle was largely attributed to misconceptions amongst the medical team. We observed that some of our bedside staff routinely checked residuals in the setting of abdominal distension, emesis or other clinical symptoms. Adherence to this measure improved after the continued education of bedside staff with evidence-based recommendations on checking gastric residuals. Similarly, there was inconsistency in the timely removal of central lines amongst providers, some of whom believed that the line should be removed once full feeding volume at 140 mL/kg/day was achieved. This was addressed by continued education as well as by providing direct reminders to the concerned provider when it was time for central line removal at 100 mL/kg/day of feeding volume.

## 4. Discussion

The implementation of several evidence-based interventions through this QI initiative reduced the NEC rate among VLBW infants by 60% over two years. The greatest reduction was observed with improved compliance for three key drivers: 1. adherence to unit feeding protocol, 2. avoiding the routine checking of gastric residuals and 3. the removal of central lines at 100 mL/kg/day of feeding volume. Our results reinforce the importance of the standardization of practices aimed at improving intestinal perfusion, limiting gut injury and mitigating the risk of infection. We found that consistency in care such as strict adherence to our unit’s feeding protocol and the continued education of healthcare providers and nursing staff led to improved outcomes.

The role of human milk in NEC prevention is well documented in the literature. In a previous retrospective review following the introduction of DHM in our unit, we observed a statistically significant reduction in the overall incidence of NEC (5% vs. 13%; *p* = 0.04) and stage III NEC (3% vs. 10%, *p* = 0.04). Even though studies have revealed variable benefits of DHM in the prevention of surgical NEC [34], our results could be explained by our lower-than-average exclusive breastfeeding rate. Following this review, we did, however, observe inconsistencies in the initiation of DHM due to delays in obtaining consent and misconceptions regarding its use among mothers, which led to a gradual increase in our rate of NEC to 7%. We also noticed that the use of MOM in our population continued to be low. Through this quality improvement initiative, where we educated the NICU staff to obtain early DHM consent and seek lactation consults to increase the use of MOM, we were able to increase both MOM availability and the DHM consent rate (when MOM was not available) to 100% in the second PDSA cycle. Though not directly measured in this QI project, the increased use of DHM promoted the earlier initiation of enteral nutrition and a faster return to birth weight in our infant cohort [30].

Adherence to our unit’s feeding protocol increased from 68% to 83% through the continued education of medical providers and bedside staff, investigating factors contributing to deviation and addressing misconceptions in real time. Al Shaikh et al. observed a reduction in the incidence of NEC from 5.6% to 1.9% across two NICUs with the increased use of MOM, improved compliance with feeding regimens, the standardization of feeding intolerance management and conservative feeding during blood transfusions and PDA treatment [35]. The investigators achieved the standardization of feeding protocols across the two units by educating bedside staff, providing updates and ensuring sustainability by facilitating group discussions and identifying potential contributing factors. Nathan et al. achieved an 83% reduction in the incidence of NEC across three NICUs following the implementation of a standardized feeding protocol and strategies to increase the use of HM [20]. Similarly, Stefanescu and colleagues implemented a comprehensive nutritional bundle to standardize parenteral and enteral nutrition initiation, advancement and weaning, incorporating the use of DHM when MOM was not available. Their NEC rate declined from 16.67% to 6.78% (*p* = 0.07), and there was a reduction in the mortality rate from 15.6% to 1.6% (*p* = 0.006) following the implementation of the bundle [16]. These findings highlight the importance of QI in the standardization of feeding practices, an evidence-based intervention in NEC prevention.

The association between infection prevention measures such as central line removal, enteral tube replacement and limiting the use of antacids has been demonstrated in several retrospective reports [33,36]. These infection prevention measures decrease the incidence of NEC by several proposed mechanisms. Central line removal removes a source of infection and break in skin integrity. Enteral tubes have been shown to be heavily colonized with pathogenetic bacteria, and the frequent checking of residuals may further increase bacterial counts. For these reasons, the regular replacement of feeding tubes and the elimination of routine gastric residual checking are recommended. Antacids decrease gastric acidity which is needed to kill bacteria and maintain a normal intestinal microflora. These practices were in place in our NICU at the start of this initiative; however, compliance was inconsistent, especially in regard to timely central line removal. This QI project, therefore, served to reinforce these practices to improve outcomes. All key drivers of our project have become standard care practices in our NICU since the implementation of these interventions.

Our key drivers are easily generalizable to other units. The use of breast milk is widely promoted in NICUs across the country. Providing lactation support and equipment to new mothers is a simple and effective way to improve breast milk provision. Similarly, the education of mothers regarding the use of donor human milk and the early initiation of enteral nutrition is another simple measure for increasing human milk use and potentially decreasing the incidence of NEC.

Standardized feeding protocols with guidelines on the initiation, advancement and fortification of feeds are in practice in most centers. What needs to be emphasized is continued adherence to these regimens. We found that deviations from the feeding algorithm mostly occurred in the setting of feeding intolerance. Though addressed on a case-by-case basis, we did not have standardized guidelines for the management of feeding intolerance. Al Shaikh and colleagues implemented a feeding intolerance algorithm as a driver following a root cause analysis to minimize such divergences. A feeding intolerance algorithm would further guide standardized management and prevent inconsistencies in care [37]. Similarly, the use of specific feeding algorithms during blood transfusions and PDA treatment is another important driver of NEC bundles [21].

Our study has several limitations given its observational nature with a small sample size. As a quality improvement project, we did not obtain individualized patient data or include a control group. Our balancing measure of adherence to checklist data demonstrated improvement for all five variables; however, only central line removal was found to be statistically significant. This limits conclusions about causality, and our observation of the decreased incidence of NEC could therefore be based solely on chance. We did not monitor compliance with some process measures such as delayed cord clamping, NGT replacement and avoiding the use of antacids either because of limited documentation or because these practices were universally observed in our NICU. As with any other QI project, the presence of other unmeasured confounders may have led to a reduction in the NEC rate. Given the nature of this initiative, it was not possible to control all variables. Furthermore, the concurrent implementation of multiple interventions also meant that it was not possible to study the extent of each intervention’s contribution to the outcome. Therefore, the relationship between the interventions and outcome is associational rather than causational.

## 5. Conclusions

The implementation of practice guidelines to increase the use of human milk, promote adherence to standardized feeding regimens, minimize infection risk and improve antibiotic stewardship led to an increased adherence to accepted practices and a decline in the incidence of NEC amongst our VLBW infants. These interventions are evidence-based, low-risk and cost-effective, and they can be easily adopted by other units. The continued implementation of NEC prevention strategies and analyses of outcomes is needed to standardize care and ensure long-term sustainability.

## Figures and Tables

**Table 1:** Demographic characteristics of included patients. N = 74, SGA—small for gestational age.

Variable		Number (Percent)
N = 74		
Sex	Male	35 (47.2)
	Female	39 (52.8)
Maternal race	Non-Hispanic Black	69(93.2)
Antenatal steroids		63(85.1)
Birth weight, g (mean ± SD)		1123 ± 287
Weight percentile	SGA	17 (22.9)
Gestational age, weeks (mean ± SD)		29.1 ± 2.9
Gestational age	23–27 + 6 weeks	20 (27)
	28–31 + 6 weeks	38 (51.4)
	>32 weeks	16 (21.6)

**Table 2:** Adherence to NEC bundle checklist items during PDSA cycles 1 and 2.

No.	Checklist Item	PDSA 1 (N = 38)	PDSA 1 (%)	PDSA 2 (N = 36)	PDSA 2 (%)	*p* Value
1	DHM consent	37	97%	36	100%	*p* = 1.00
2	Adherence to feeding protocol	26	68%	30	83%	*p* = 0.221
3	No checking of residuals	29	76%	34	94%	*p* = 0.062
4	Discontinue Abx < 48 h	31	81.6%	32	88.8%	*p* = 0.376
5	Central line removal	27	71%	33	91.6%	*p* = 0.023 *
	Overall adherence		78%		91.6%	*p ≤* 0.05 *

* Statistically significant for *p* < 0.05.

## Data Availability

Data are available upon reasonable request. The data that support the findings of this study are available on request from the corresponding author. The data are not publicly available due to privacy or ethical restrictions.
